# Snail control as a crucial approach to schistosomiasis elimination: evidence from the People’s Republic of China

**DOI:** 10.1186/s40249-025-01281-0

**Published:** 2025-02-21

**Authors:** Shan Lv, Jing Xu, Yin-Long Li, Zi-Ping Bao, Li-Juan Zhang, Kun Yang, Dan-Dan Lin, Jian-Bing Liu, Tian-Ping Wang, Guang-Hui Ren, Bo Zhong, Yi Dong, Li Cai, Li-Yong Wen, Zhi-Hua Jiang, Zhuo-Hui Deng, Han-Guo Xie, Shi-Zhu Li, Robert Bergquist, Jürg Utzinger, Xiao-Nong Zhou

**Affiliations:** 1 National Key Laboratory of Intelligent Tracking and Forecasting for Infectious Diseases, National Institute of Parasitic Diseases at Chinese Center for Disease Control and Prevention (Chinese Center for Tropical Diseases Research); Key Laboratory on Parasite and Vector Biology, National Health Commission; WHO Collaborating Centre for Tropical Diseases; National Center for International Research on Tropical Diseases, Ministry of Science and Technology, Shanghai, People’s Republic of China; 2 Jiangsu Institute of Parasitic Diseases, Wuxi, People’s Republic of China; 3 Jiangxi Institute of Parasitic Diseases, Nanchang, People’s Republic of China; 4 Hubei Provincial Center for Disease Control and Prevention, Wuhan, People’s Republic of China; 5 Anhui Institute of Parasitic Diseases, Hefei, People’s Republic of China; 6 Hunan Institute of Schistosomiasis Control, Yueyang, People’s Republic of China; 7 Sichuan Provincial Center for Disease Control and Prevention, Chengdu, People’s Republic of China; 8 Yunnan Institute of Endemic Diseases Control and Prevention, Dali, People’s Republic of China; 9 Shanghai Municipal Center for Disease Control and Prevention, Shanghai, People’s Republic of China; 10 Zhejiang Provincial Center for Schistosomiasis Control, Hangzhou Medical College, Hangzhou, People’s Republic of China; 11 Guangxi Zhuang Autonomous Region Center for Disease Prevention and Control, Nanning, People’s Republic of China; 12 Guangdong Provincial Center for Disease Control and Prevention, Guangzhou, People’s Republic of China; 13 Fujian Provincial Center for Disease Control and Prevention, Fuzhou, People’s Republic of China; 14 Geospatial Health, Brastad, Sweden; 15 Swiss Tropical and Public Health Institute, Allschwil, Switzerland; 16 University of Basel, Basel, Switzerland

**Keywords:** *Oncomelania hupensis*, Nationwide survey, Habitat, Accumulated snail-infested range, Elimination, Schistosomiasis

## Abstract

**Background:**

Asian schistosomiasis is projected to be eliminated by 2030 according to World Health Organization road map for neglected tropical diseases 2021–2030. Snail control is an important measure but has not yet been systematically evaluated at a country scale. Here, we report the findings from a nationwide survey to demonstrate the dynamics of *Oncomelania* and its potential role in transmission interruption of schistisomiasis in the People’s Republic of China (P.R. China).

**Methods:**

Between March 2016 and December 2017, we conducted a nationwide census on *Oncomelania* snail habitats in P.R. China. All historically recorded snail habitats were identified and reviewed. Information on habitat attributes, including the infestation of snails, was collected. The shape of habitats was determined using global positioning system and geographical information system technologies. The relationship between snail control and schistosomiasis elimination was established in 378 endemic counties. The comparison of accumulated snail-infested range (ASR) and the median ratio of eliminated ASR between the transmission-interrupted and endemic counties was tested by a non-parametric test (Mann–Whitney) with a significance level of 0.05.

**Results:**

Overall, 15,377.7 million m^2^ of potential snail habitats with a total of 356,550 snail habitats were identified in P.R. China. The ASR amounted to 86.0% of the total area. Most of the ASR (94.9%) and habitats (68.5%) were distributed in the middle and lower reaches of the Yangtze River. Snail habitats were found up to an altitude of 2859 m above the mean sea level. By 2017, 85.1% of habitats (73.0% of the ASR) had been eliminated with almost half of them eliminated between 1965 and 1982. The elimination of snail habitats promoted transmission interruption of schistosomiasis, but showed variable patterns in different landscapes. The ratio of eliminated ASR was 99.6 and 91.4% in water network and hilly areas, respectively, while it was only 64.8% in marshland areas, particularly in Hunan and Jiangxi where the two largest freshwater lakes of P.R. China are located. Marshland habitats were seen as the most difficult for transmission interruption, which calls for additional control measures in these settings.

**Conclusions:**

Our results support recent recommendations by the World Health Organization to implement snail control and demonstrate that schistosomiasis elimination can be achieved. The nationwide, high-resolution map of *Oncomelania* snail habitats in P.R. China will support further efforts to eliminate schistosomiasis.

**Graphical Abstract:**

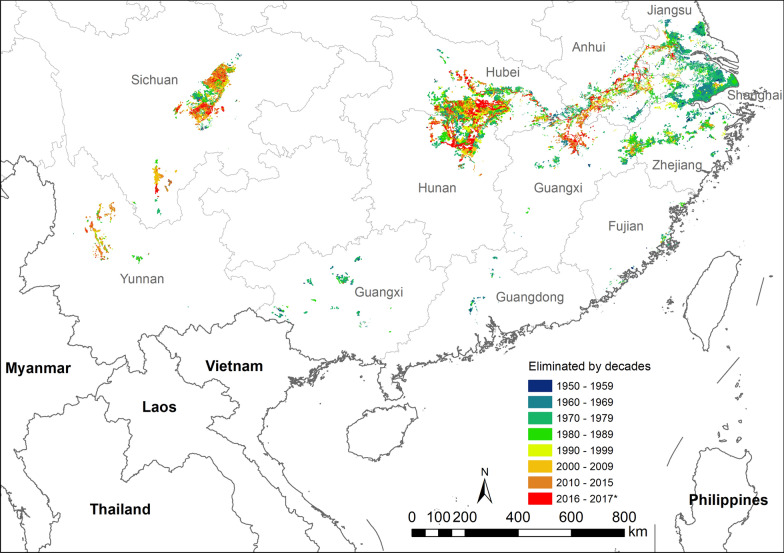

**Supplementary Information:**

The online version contains supplementary material available at 10.1186/s40249-025-01281-0.

## Background

Human schistosomiasis, caused by five major species of the trematode *Schistosoma*, is endemic in 78 countries, and more than 800 million people worldwide live in areas endemic for schistosomiasis [1]. According to the road map for neglected tropical diseases (NTDs) 2021–2030 issued by the World Health Organization (WHO), all endemic countries are expected to achieve the elimination of schistosomiasis as a public health problem and 25 countries should achieve transmission interruption by 2030 [2]. Asian schistosomiasis is endemic in five countries, i.e., Cambodia, the People’s Republic of China (P.R. China), Indonesia, Laos, and the Philippines. According to the WHO’s NTDs roadmap 2021–2030, Asian schistosomiasis will be eliminated by 2030.

Schistosomiasis is a parasitic disease with a life cycle that requires a snail intermediate host [3]. For this reason, snail control is an important means of control. The 65th World Health Assembly (WHA) in 2012 proposed taking advantage of programmes outside the immediate health area, such as general improvement of the environment, to accelerate the interruption of schistosomiasis transmission by elimination of the snail intermediate host [4], a message further underscored at the 70th WHA in 2017 [5]. In response, the WHO has refocused on snail control, a strategy that was largely abandoned in 1985 [6] and has recently published guidelines for laboratory and field testing of molluscicides in the control of schistosomiasis [7].

P.R. China was once one of the most affected countries with an estimated 11.8 million cases of schistosomiasis in the mid-1950s [8]. Meanwhile, more than 95% of endemic counties have achieved transmission interruption (TI) or elimination in P.R. China [9]. Two hundred and one out of 453 endemic counties had become free of *Oncomelania* spp. snails and the nationwide snail-infested areas declined from 8.2 billion m^2^ in the mid-1950s to 3.6 billion m^2^ in 2020 [9, 10]. The simultaneous decline in schistosomiasis prevalence and snail-infested areas implies that snail control has played an essential role.

Snail control in P.R. China can be stratified into chemical control using molluscicides, environmental modification as well as physical removal [11]. Although the application of molluscicides was able to reduce the snail density, and hence, the risk of schistosomiasis transmission, snail elimination was rarely achieved by molluscicides alone [7, 8]. Of note, *Oncomelania* *hupensis* are amphibious. The female snails lay eggs into wet soil that is washed into freshwater bodies, often during flooding events, where they hatch. Schistosomiasis transmission is therefore sensitive to hydrological events. Water conservancy projects against flooding can change the hydrological conditions through attenuation of fluctuation of water levels through the use of artificial reservoirs, and by reducing runoff downstream through the construction of dams. Both of these measures have a devastating impact on snail populations [12–14]. Development of agricultural irrigation systems is also playing an important role in governing the distribution of *O. hupensis*, as artificial barriers were added to original water networks [15]. Other environmental modifications include replacing old irrigation ditches with new cemented canals; construction of water storages for fishing and irrigation, change of land use, among others [11].

The Yangtze River basin, which is the major endemic region of schistosomiasis, is home to many water conservancy developments [16]. In 1949–1957, embankments were strengthened along the Yangtze River to deter flooding and construction of flood diversion and storage systems were undertaken. Embankment to a large extent blocked the spread of *O. hupensis* from the river and lakes to irrigated region, which facilitated the elimination of snails in the latter. Over the next 20 years, from 1958 to 1977, many small reservoirs and irrigation systems abruptly emerged to ensure the water supply for agriculture, accompanied by land reclamation. The snails disappeared soon after dam constructions [13, 14], which was in contrast to the impacts of dam construction on most other *Schistosoma* species [1]. The fastest elimination of snail habitats occurred in the 1960s and 1970s [17], which coincided with the development of agriculture and water conservancy [18, 19].

In this paper, we present results from a nationwide snail survey conducted between March 2016 and December 2017. We mapped the spatiotemporal dynamics of *O. hupensis* distribution and explored the relationship between snail control and schistosomiasis elimination at a national scale for the first time.

## Methods

### Study area and habitat definitions

All counties (*n* = 453) historically known to be endemic for schistosomiasis in 12 provincial-level administrative divisions (PLADs) of P.R. China were included in the study (Figure S1). A habitat is defined as a relatively independent physical environment infested with *O. hupensis*, with adjacent habitats separated by obvious barriers, or their ecological features are essentially different. A habitat can be an artificial man-made environment (e.g., irrigation ditch or channel), or naturally formed (e.g., marshland). Since many habitats remained from the 1950s when the national schistosomiasis control programme was launched, we accepted conventional definitions for the purpose of maintaining the integrity of data. We defined an extinct habitat when *O. hupensis* were not found in two or more consecutive years. Otherwise, the habitat was considered as extant.

A habitat is not necessarily fully infested by *O. hupensis*. To consider this aspect, we introduced the term “snail-infested range” (SIR) to indicate the actual distribution of snails in the habitat. Hence, a SIR can be less or equal to the overall area. In addition, since the actual distribution of *O. hupensis* in a habitat may change or shift from one year to another, the term “accumulated snail-infected range” (ASR) was employed to indicate the maximum distribution range in the habitat by overlapping annual actual distribution ranges from the time that *O. hupensis* had been discovered in the habitat.

### Data collection and analysis

The national protocol of snail survey was prepared by the National Health Commission and the National Institute of Parasitic Diseases (NIPD) at Chinese Center for Disease Control and Prevention (China CDC). National training sessions were held with the principal investigators at the province level and they in turn trained the staff from local official centres for disease control and prevention (local CDCs) or institutes of schistosomiasis control (ISCs). The habitat survey was performed by skilled staff. The principal investigators sampled and visited 20% of the counties for quality control, (i) to check data completeness in database, and (ii) to confirm the status (extant or extinct) of habitats.

All habitats documented by annual records were registered in the first step of this investigation. The annual records could date back the 1950s and kept in the local agencies of schistosomiasis control. Each habitat was coded by a unique 13-digit identification number that included 2 digits each for each spatial step (PLAD, city, county, township and community) plus a 3-digit serial number. The information pertaining to the habitats were extracted from annual records, including the setting they came from (i.e. marshland, water network or mountainous/hilly landscape), habitat type (e.g., ditch, pond, marshland, etc.), habitat size (m^2^), year for first discovery and year of elimination of *O. hupensis*, initial SIR when snails were first discovered (m^2^), ASR (m^2^) and extant SIR (m^2^). Data were entered into a Microsoft Excel (Microsoft Corp., Redmond, WA, USA) spreadsheet, double-checked at the county level and pooled into a Microsoft Access (Microsoft Corp., Redmond, WA, USA) database at the national level. Statistical analysis was performed by SPSS version 19.0 (IBM Corp., Armonk, NY, USA).

The spatial habitat data were obtained by global positioning system (GPS) and organized by a geographical information system (GIS). Briefly, small habitats were drawn up by tracing with hand-held GPS in WGS1984. For larger habitats, the coordinates of four or more key boundary points were obtained by GPS with habitat shapes determined in GIS based on remotely sensed images (Figure S2). Each habitat shape file was referred to by its unique 13-digit ID. Habitats destroyed by a land use change approach were mapped according to previous annual records by senior staff. The attribute and shape data of habitat were linked by their unique ID in ArcGIS version 10.1 (ESRI, Redlands, CA, USA).

The occurrence of *O. hupensis* was confirmed by standardized sampling procedure, namely systematic sampling and environmental sampling. The description of the methods has been published elsewhere [20].

The elevation data covering the study area were obtained from a digital elevation model (DEM; spatial resolution: 90 × 90 m) of Shuttle Radar Topography Mission (SRTM) (http://srtm.csi.cgiar.org/srtmdata/). A grid with slope data was produced based on DEM and then a merged spatial shape without steep depressions across the study area was created in ArcGIS 10.1. The habitats could then be plotted in three dimensions (3-D) by ASR, elevation and slope in Origin version 9.1 (OriginLab Corp., Northampton, MA, USA). We explored habitat clustering at the elevation level. We first divided the elevation range into subgroups every 10 m from 0 m. The frequency of habitats in each subgroup was calculated. A cluster was consist of 10 or more consecutive subgroups with a frequency of more than 250 habitats.

We also noted information on TI in the endemic counties, defined as absence of infection in humans, animals or *O. hupensis* for at least 5 years [21]. The ratio of eliminated ASR was defined as the proportion of the eliminated snail-infested areas in the ASR by the year when a county achieved TI criteria or by 2016. The relation between TI and the ratio of eliminated ASR was analysed. Comparison between TI counties and non-TI counties using non-parametric test (Mann–Whitney) with a significance level of 0.05 was performed in SPSS.

## Results

### Spatial patterns

**Fig. 1:**
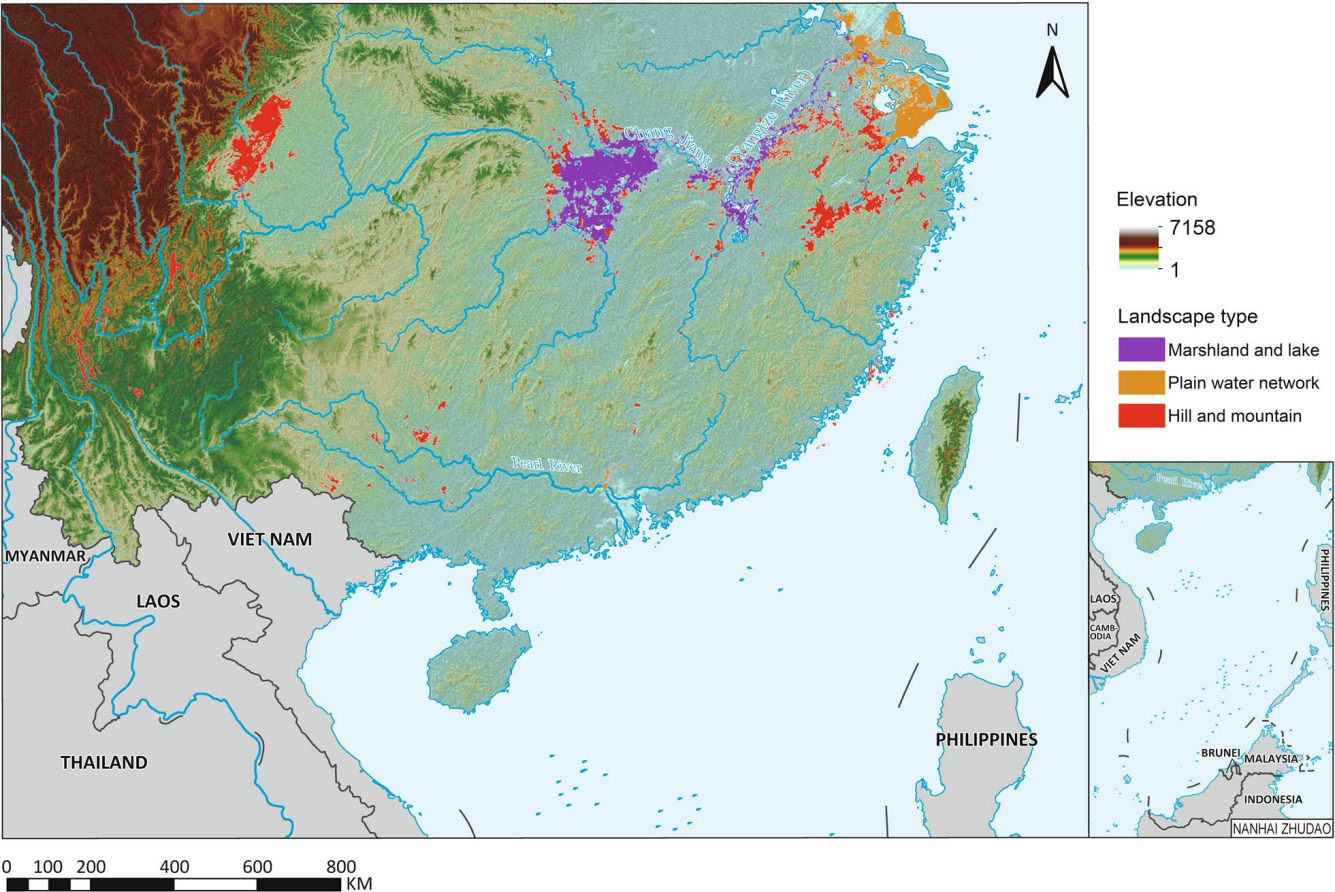
The distribution of snail habitats by landscape types. Map approval No.: GS (2025)0290

Snail habitats were overwhelmingly concentrated in the drainage area of the Yangtze River and in the neighbouring hills in Zhejiang province (Fig. 1). Only 1.4% of the snail habitats (1.1% ASR) were found in Fujian, Guangdong and Guangxi (Table 1). Along the Yangtze River, the habitats showed different distribution patterns in its upper and lower reaches. Approximately 30.1% of habitats (4.0% ASR) were distributed in the upper reach, while 68.5% of habitats (94.9% ASR) were in the middle and lower reaches. Furthermore, there was an obvious separation between the upper and lower reaches of the Yangtze River. The snail habitats in the latter were found to be mainly distributed along the Yangtze River, its major branches, and in the lakes or plain water networks connected to the these water systems. However, the habitats in the hills and mountainous areas in the upper reaches were often found in small ditches and irrigation canals beyond the major rivers. Similar patterns were also observed in the hilly areas around the flooding plain in the middle and lower reaches.

**Table 1:** Distribution of *Oncomelania* spp. snail habitats by province in P.R. China

PLAD	All habitats				Extant habitats		REA (%)	Year of TI
	No. of habitats	Area (million m^2^)	Average size (m^2^)	ASR (million m^2^)	No. of habitats	SIR (million m^2^)		
Sichuan	87,846	417.9	4758	296.0	19,314	45.8	84.5	2017
Jiangsu	77,149	1430.4	18,540	1,208.0	641	27.9	97.7	2019
Zhejiang	56,337	911.5	16,180	644.5	1315	1.0	99.9	1995
Hubei	43,422	4341.4	99,980	3953.9	16,718	721.1	81.8	–
Shanghai	25,254	257.8	10,209	165.6	90	0.03	99.9	1985
Anhui	22,757	1621.5	71,252	1410.0	4830	265.0	81.2	-
Yunnan	19,651	416.4	21,187	229.2	6440	20.3	91.1	-
Hunan	9683	3304.1	341,224	3145.5	1707	1,663.6	47.1	-
Jiangxi	9616	2510.5	261,077	2,023.0	2175	832.3	58.9	-
Guangxi	2326	29.0	12,481	26.8	10	0.06	99.8	1988
Fujian	1721	34.2	19,859	26.5	14	0.02	99.9	1987
Guangdong	788	103.0	130,759	100.6	0	0.0	100.0	1985
Total	356,550	15,377.7	43,129	13,229.6	53,254	3,577.2	73.0	-

*ASR* accumulated snail-infested range, *SIR* snail-infested range, *PLADs* provincial-level administrative divisions, *REA* ratio of eliminated area, *TI* transmission interruption

The marshland habitat class is exclusively located along the lower parts of the Yangtze River, its major branches and the connected lakes. Although the habitats in these areas were only responsible for 13.5% of the total snail habitats, they provide considerable risk for snail infestation that accounted for 68.3% in the country (Table 2). The hilly type of habitats is predominantly seen in the mountainous areas of Sichuan and Yunnan provinces. It also exists around marshlands in the lower reaches of the Yangtze River, as well as in the hilly regions in Zhejiang, Fujian and Guangxi. This type of connected water network was mainly seen in the Yangtze delta, including Shanghai, Jiangsu and Hangzhou Bay in Zhejiang province. Although the number of habitats in the mountainous areas and the water network regions accounts for 86.5% in the country, the average size of habitats is only 7.3% of those in the marshlands. Accordingly, the overall area of habitats in the mountainous areas and the water network regions were only 20.8% and 10.9%, respectively.

**Table 2:** Distribution of snail habitats by landscape types in P.R. China

Landscape type	All habitats				Extant habitats		REA (%)
	No. of habitats	Area (million m^2^)	Average size (m^2^)	ASR (million m^2^)	No. of habitats	SIR (million m^2^)	
Marshland	48,219	10,496.0	217,674.5	9603.3	18,124	3379.9	64.8
Water network	125,834	1674.8	13,309.5	1381.8	423	4.8	99.6
Hilly areas	182,497	3206.8	17,572.0	2244.5	34,707	192.5	91.4
Total	356,550	15,377.6	43,129.1	13,229.6	53,254	3577.2	73.0

*ASR* accumulated snail-infested range, *SIR* snail-infested range, *REA* ratio of eliminated area

### Temporal patterns

**Fig. 2:**
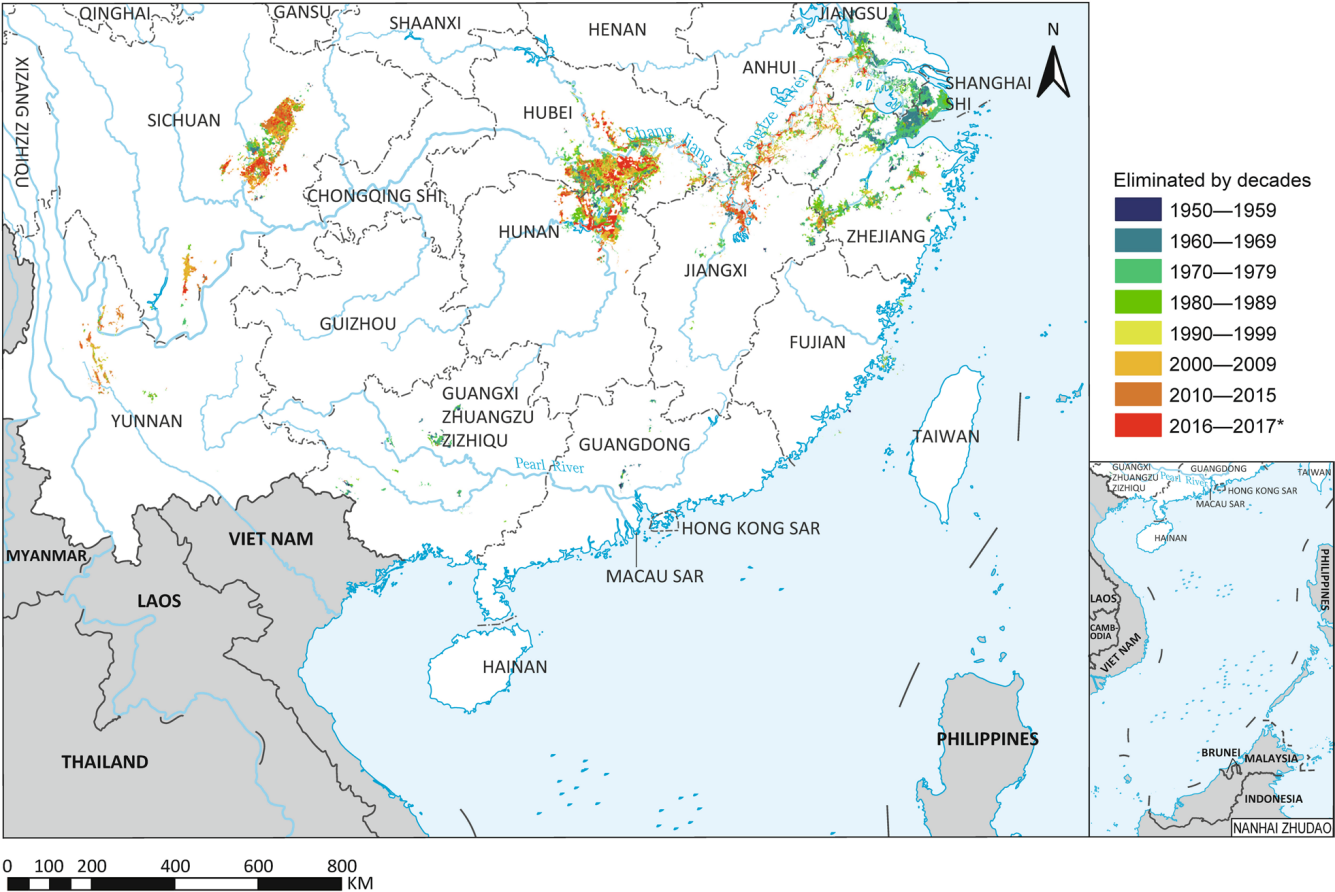
The spatiotemporal dynamics of snail habitats. The 12 endemic provinces of P.R. China are named in the map. Map approval No.: GS (2025)0290

A total of 356,550 *O. hupensis* habitats with an overall area of 15,377.7 million m^2^ were identified through the census. This corresponds to an ASR of 13,229.6 million m^2^ in P.R. China (Table 1). By 2017, 85.1% habitats (73.0% ASR) had been eliminated. The early eliminated habitats were clustered in the eastern water network region, including Shanghai, Zhejiang and Jiangsu (Fig. 2). By the end of 1989, 97.1% of the habitats (92.5% of ASR), had been eliminated in the three PLADs. Other early eliminated habitats were observed in Fujian, Guangdong and Guangxi beyond the Yangtze River basin.

**Fig. 3:**
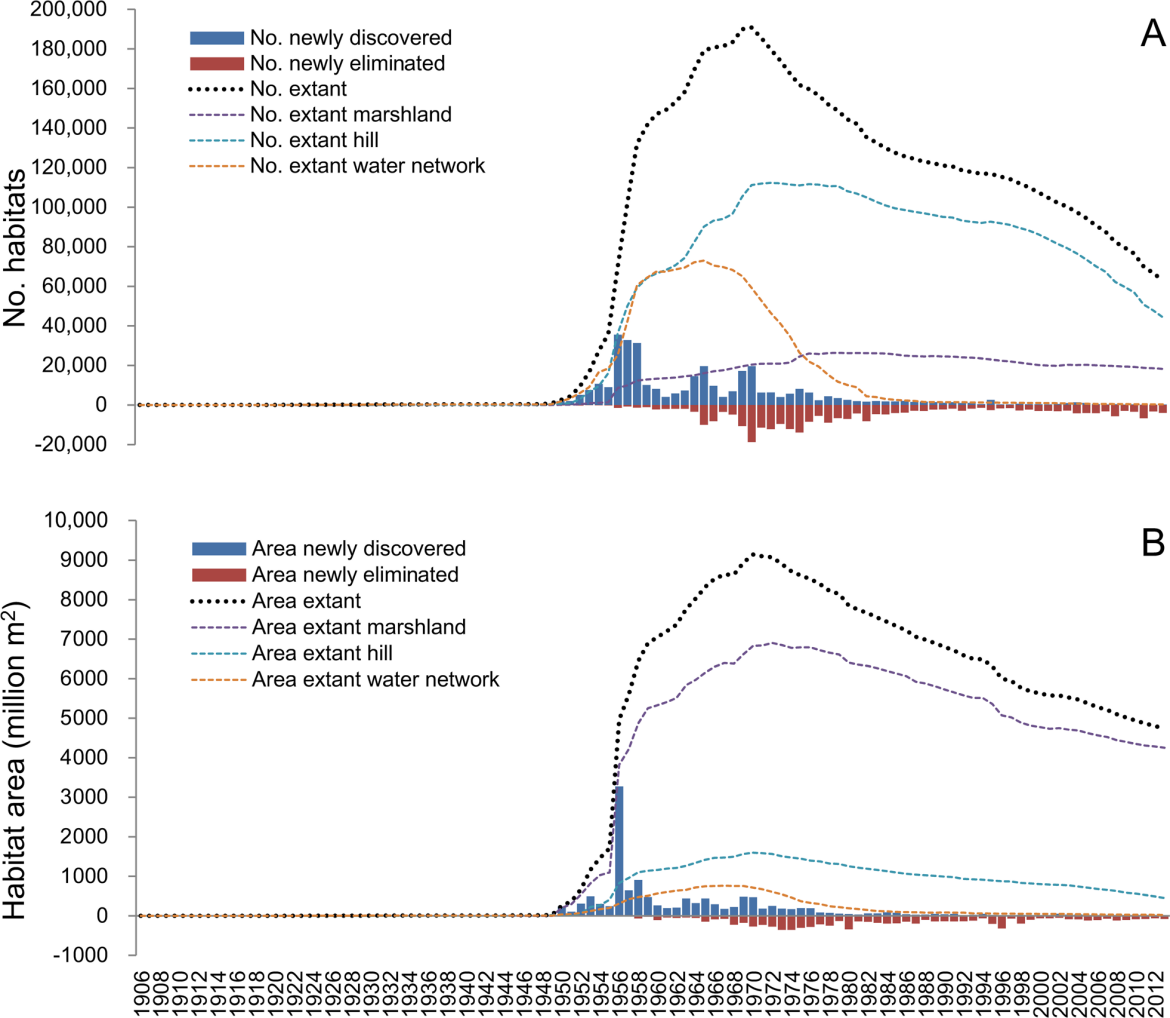
The temporal pattern of snail habitats (**A**) and snail-infested range (**B**)

The first record of a habitat infested by *O. hupensis* dates back to 1906, located in Sichuan province. The first records of *Schistosoma*-infected snails in Shanghai and Zhejiang date back to 1930. Both the number of newly discovered habitats and ASR sharply increased since 1956 when the first nationwide survey on schistosomiasis was conducted (Fig. 3A, B). There were another two obvious peak years for newly discovered habitats (1965 and 1970). Meanwhile, the elimination peak of habitats and ASR appeared in 1970 and 1973, respectively. The speed of elimination declined around 1994, but increased again after 2000 although the magnitude was lower than that in the 1960s and 1970s. About 47.1% of snail habitats were eliminated between 1965 and 1982. The ASR decreased by 49.3% in the corresponding period.

The peaks of extant habitat number and ASR occurred in 1970 with amounts of 190,768 and 9141.6 million m^2^, respectively. Snail habitats declined steadily thereafter. The dynamic of habitat change and distribution of ASR over time in the three landscape types investigated showed striking differences (Fig. 3A, B).

The peaks of extant habitats and ASR in water network region occurred in 1965 and 1967, respectively, followed by mountainous region (1972 and 1970) and marshlands (1979 and 1972). The extant habitats and ASR in water network region had rapidly decreased from the peaks by 86.1 and 74.9% by 1980. The number of extant snail habitats and ASR in the hilly/mountainous areas had declined more slowly between 1972 and 2000, while the trend was accelerated from 2000 onwards. The amount of extant habitats and ASR in marshlands was reduced by 30.5 and 47.6% over the past 40 years.

### Topographical pattern

**Fig. 4:**
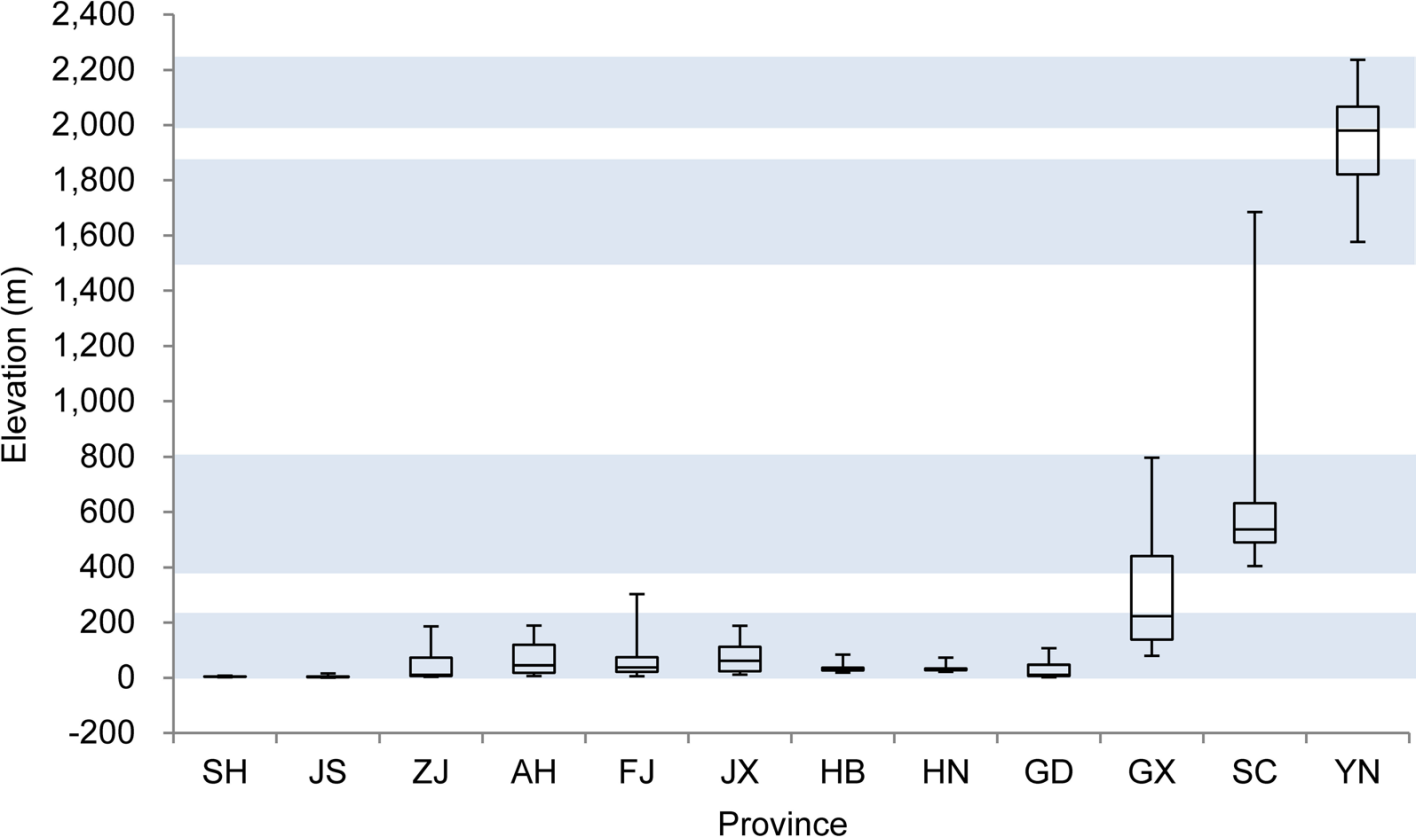
Boxplot of habitat elevation in different provinces. *SH* Shanghai, *JS* Jiangsu, *ZJ* Zhejiang, *AH* Anhui, *FJ* Fujian, *JX* Jiangxi, *HB* Hubei, *HN* Hunan, *GD* Guangdong, *GX* Guangxi, *SC* Sichuan, *YN* Yunnan. The boxplot whiskers represent the 2.5 and 97.5%. The *blue belts* indicated four clusters as described in text

About 95% of the habitats were located below 1755 m above the mean sea level (msl). The highest altitude occurrence of *O. hupensis* was reported from Zhaojue county in Sichuan province (2859 m). The median elevation was 33 m. We identified four main groups across P.R. China (Fig. 4). The first group consisted of habitats ranging from 1 to 230 m and included all habitats in the marshlands and water networks, as well as those in the mountenous/hilly regions around the marshlands. The second group was located at altitutdes between 370 and 790 m. Most habitats were distributed in the Sichuan basin and the rest were from the hilly areas in Guangxi. The third group were at altitudes of 1470–1840 m. These habitats were located in southern Sichuan and Yunnan provinces. The fourth group were between 1960 and 2210 m. Most of them were concentrated in Yunnan province.

**Fig. 5:**
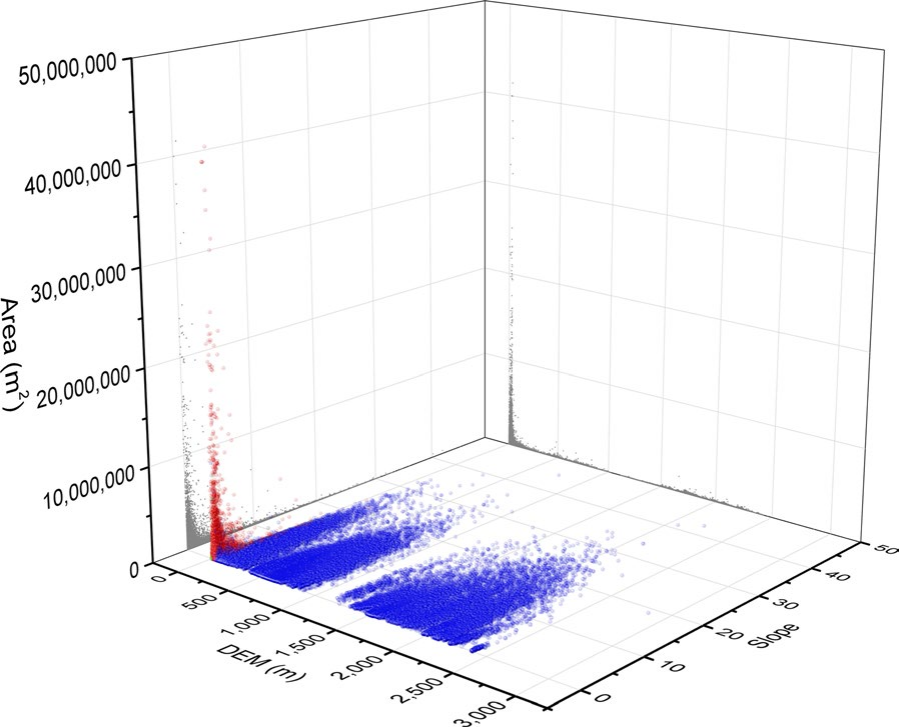
3-D scatter plot of habitats by accumulated snail-infested area, elevation and slope. The balls in *blue*, *red* and *green* represent the habitats in hills, marshland and plain water network, respectively. 2-D scatter plot was also projected using *grey colour*

The distribution of habitat slope was typically skewed (Fig. 5). The skewness coefficient was 3.8. The slope ranged from 0° to 45.2° degree with a median of 0.6°, where 95% habitats had a slope below 7.5°. The steepest habitat was in Tianquan county, Sichuan province. We employed the method of kernel density to extract the plains (Figure S3) and found that 72.0% habitats (87.2% ASR) were located in plain areas. Furthermore, about 94.8% of the ASR in the marshlands and 96.6% in the water network areas were distributed in the plains, while 48.8% ASR in hilly areas were in the plains.

### Relationship between snail elimination and schistosomiasis transmission interruption

**Fig. 6:**
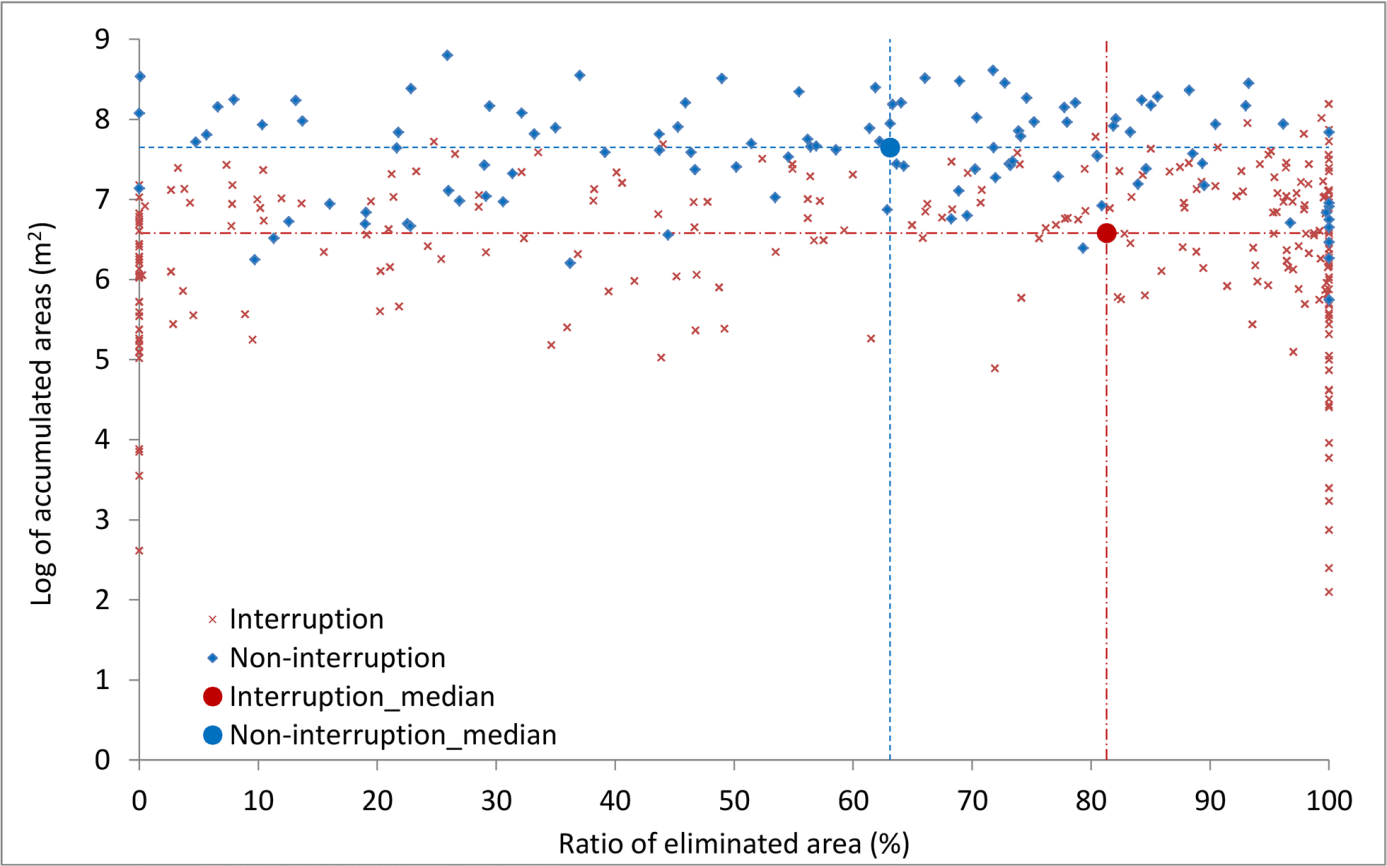
Relation between transmission interruption and ratio of eliminated snail-infested area or accumulated snail-infested area

A total of 378 out of 453 *S. japonicum*-endemic counties were included for the analysis to explore the contribution of snail elimination to TI. The rest of 75 counties were excluded due to incomplete records when the last infections in human, domestic animal or snail occurred. Of the 378 included counties, 265 achieved the TI criteria by 2017 (Fig. 6). The remaining 113 counties were endemic for schistosomiasis. The ASR range of TI counties was from 126 m^2^ to 696.2 million m^2^ with a median of 3.8 million m^2^. That of the endemic counties were from 560,089 m^2^ to 655.3 million m^2^ with a median of 44.7 million m^2^. The median ASR of TI counties was significantly lower than that of endemic counties (*P* < 0.05). The median ratio of eliminated ASR was 63.1% in endemic counties, while that in TI counties was 81.3%, which was significantly higher than the former (*P* < 0.05).

The ratio of eliminated ASR was also calculated by province (Table 1). Five PLADs that maintained the TI status over a 20-year period (i.e. Guangdong, Shanghai, Fujian, Guangxi and Zhejiang) had eliminated ASR by 99%. Meanwhile, the first four PLADs had the smallest ASR among all 12 endemic PLADs. Two provinces that recently achieved TI criteria (i.e. Sichuan, a mountainous endemic area and Jiangsu, a plain area at the down reach of Yangtze River) eliminated 84.5 and 97.7% ASR, respectively. The ratio of eliminated ASR less than 60% was observed only in Hunan and Jiangxi provinces where the two largest freshwater lakes of P.R. China are located.

## Discussion

In P.R. China, a major feature of successful schistosomiasis control is the shrinking of snail-infested areas. Indeed, the reduction in ASR by over 99% in five provinces has helped to maintain the TI status for more than 20 years. Our results also suggest that the endemic counties with smaller ASR and/or a larger ratio of eliminated SIR are more likely to achieve TI. Such conclusions are corroborated by data from Japan, where *S. japonicum* was endemic, but the disease was eliminated in 1976 [5]. Of note, *Oncomelania* spp. snails once occurred in six separate areas in Japan. Snail control served as the major approach to interrupt the transmission of schistosomiasis. Only few foci of the Japanese snail intermediate host, *O. h. nosophora*, remain extant, and these lie in two different endemic areas (Kofu Basin and Obitsu) [22], which maintains the necessity for continued attention to schistosomiasis control in the country.

In Indonesia, the overall prevalence of schistosomiasis in humans, rats and snails was 2.6, 8.6 and 2.4% between 2008 and 2011, respectively [23]. Although more than half of old foci of *O. h. lindoensis* disappeared in some areas, several new foci were observed in conventional endemic valleys (Lindu and Napu) and even new endemic areas (Bada valley) was recently identified [23, 24]. The national baseline survey in the Philippines between 2005 and 2008 indicated that the prevalence of *S. japonicum* ranged from 0.1 to 6.3% in 23 provinces [25]. Although snail control is included as an additional measure in schistosomiasis control in the two countries, no pronounced effect was observed [26, 27]. WHO is reinforcing snail control as part of its strategic approach to achieve the target of eliminating schistosomiasis as a public health problem by 2030 [2, 6]. Snail control can be implemented where there is high prevalence.

The achievement of schistosomiasis control in P.R. China is attributed to intersectoral collaboration. Although application of chemical molluscicides conducted by the public health sector reduce snail densities, and hence, effectively prevent infections, elimination of *Oncomelania* spp. snails by means of environmental modification is more effective in a long term [11]. However, schistosomiasis control programmes for sectors other than public health were terminated in recent years due to very low prevalence [28], which may be a potential trigger of resurgence of *O. hupensis*. Hence, collaboration across sectors (i.e., water conservancy, agriculture, land source management, forestry and public health), coordinated by the central and local governments, plays an essential role in the national snail control programme leading to reduced schistosomiasis transmission [29].

The snail habitats in the marshlands are the most difficult ones to deal with. The huge areas make it impossible to eliminate *Oncomelania* spp. snails by conventional measures (e.g., application of molluscicides, land reclamation or small water conservancy projects). However, recent evidence indicates that the construction of the Three Gorges dam contributed to decline of snail density in the marshlands. Both snail density and snail-infested areas in previous habitats are declining in the whole middle-lower reach of the Yangtze River [30–32]. Nevertheless, the lower water level has led to emerging new marshland ranges with the potential of becoming areas of new *O. hupensis* habitats [33]. Long-term effects are being monitored. On the other hand, infections in wild animals in these areas of marshlands are not taken into account by the national schistosomiasis control programme, which is making more challenges in reaching the elimination targets in P.R. China [34–36]. Boatmen or fishermen there have frequent contact with water in the marshland habitats, which thus might become the bridge between natural transmission in the marshlands and other sectors of the population [37]. Taken together, the success of schistosomiasis elimination in P.R. China depends on the effectiveness of snail control in the marshlands. Hence, the Chinese Government should update the strategy for schistosomiasis control in middle-lower reach of the Yangtze River.

Although the present nationwide survey involved all agencies of schistosomiasis control, and quality control guidelines were adhered to, there are still some shortcomings. First, we did not collect the year of resurgence for those habitats in which snails were found to have reappeared. Resurgence of snails in historically endemic areas occurred frequently, which challenges the consolidation of schistosomiasis transmission interruption [38]. In order to respond to the resurgence, the counties where schistosomiasis was endemic historically should maintain the capacity of surveillance and response by routine trainings. Second, missing habitats and data were inevitable since some habitats were eliminated very early and the information was not complete. There are 8199 habitats with unknown year of discovery, accounting for 1.1% ASR. Third, we followed the conventional system for classification of habitat landscape type, as used throughout much of China’s snail control history. However, some habitats around lakes might be classified into water network rather than marshland because the original marshland had been transformed into agricultural land. Such habitats are more similar to those in plain water network, as is commonly observed in the eastern part of P.R. China. Finally, we used a constant ASR of snail habitat to replace the annual real snail-infested area as the indicator for temporal pattern analysis, which might lead to an overestimation of the annual nationwide ASR of extant habitats.

## Conclusions

We present a nationwide high-resolution map of *Oncomelania* spp. snail habitats in P.R. China. This map furthers the understanding of the spatiotemporal dynamics of snail habitats and emphasizes the importance of snail control for schistosomiasis elimination, and can be readily tailored to specific landscapes. Although the ecological characteristics of *Oncomelania* spp. are different from the snail intermediate host required by other *Schistosoma* species, our results—along with the recommendations put forth by WHA in 2012 and in 2017—call for implementation of snail control as essential feature required for the elimination of schistosomiasis. Our findings suggest an evaluation of the cost-effectiveness of snail control and some complementary measures for schistosomiasis control, since lower efficacy may be observed in some large-sized habitats, which could only be influenced through large water conservancy projects.

## Supplementary Information

Additional file 1: Figure S1. Schistosomiasis-endemic counties (yellow and red) and PLADs (grey) in P.R. China. The counties in red denoted those where the transmission of schistosomiasis was not interrupted by 2017. Map approval No.: GS (2025)0290.

Additional file 2: Figure S2. Digitalization flow of habitats based on historical data.

Additional file 3: Figure S3: The plain areas (blue) distribution in the central and southern P.R. China. Map approval No.: GS (2025)0290.

## Data Availability

The data from the national survey on *Oncomelania* snail are deposited at National Institute of Parasitic Diseases at China CDC (Chinese Center for Tropical Diseases Research). The data can be accessible based on personal communication to corresponding author (lvshan@nipd.chinacdc.cn).
