# Supplementary figures and images for: Snail control as a crucial approach to schistosomiasis elimination: evidence from the People’s Republic of China
Source: Infect Dis Poverty. 2025 Feb 21;14:10. doi: 10.1186/s40249-025-01281-0 (PMC11846187; doi:10.1186/s40249-025-01281-0)

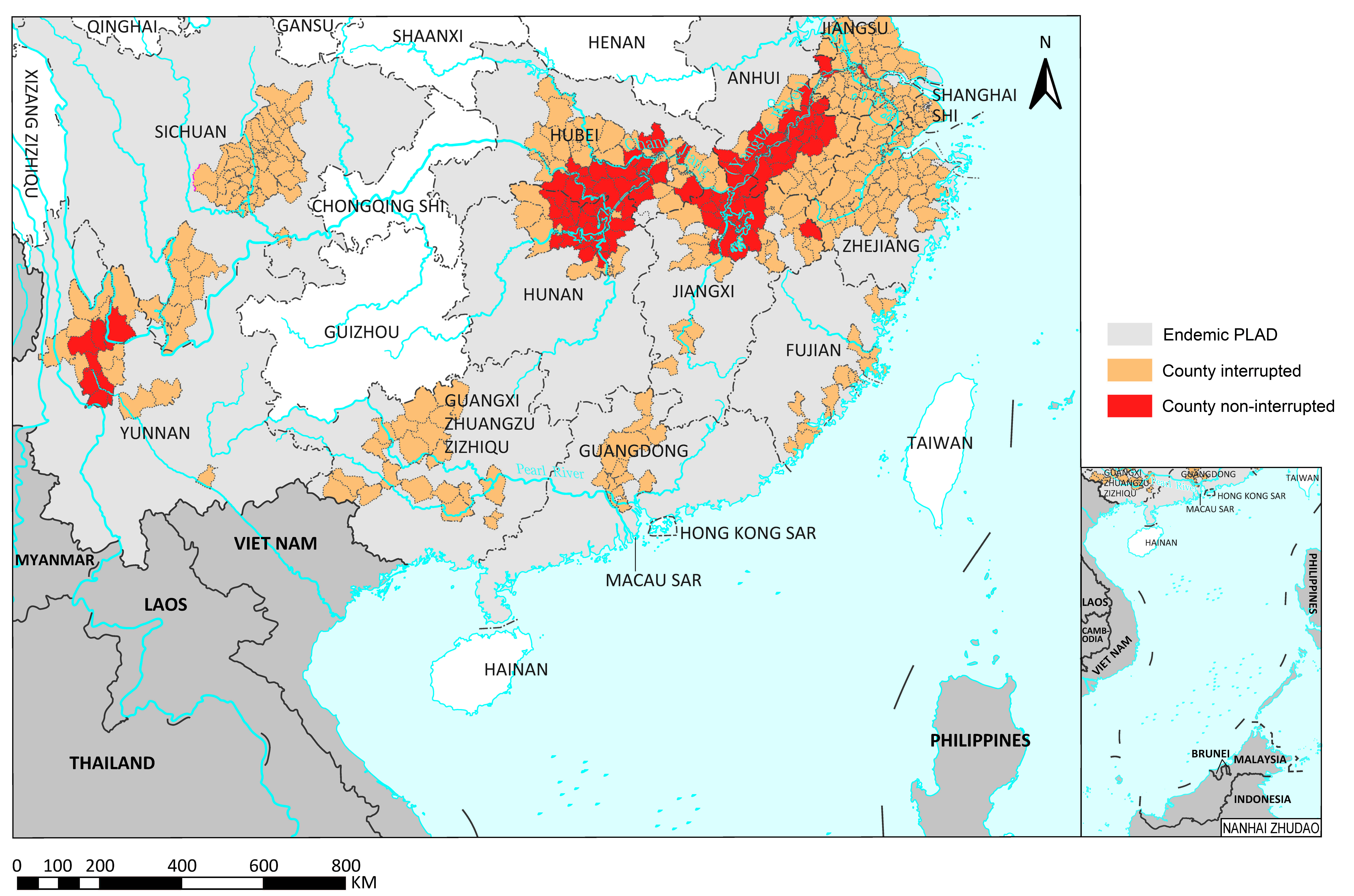

Supplement: Supplementary file 1 — Additional file 1: Figure S1. Schistosomiasis-endemic counties (yellow and red) and PLADs (grey) in P.R. China. The counties in red denoted those where the transmission of schistosomiasis was not interrupted by 2017. Map approval No.: GS (2025)0290. [file 40249_2025_1281_MOESM1_ESM.jpg]

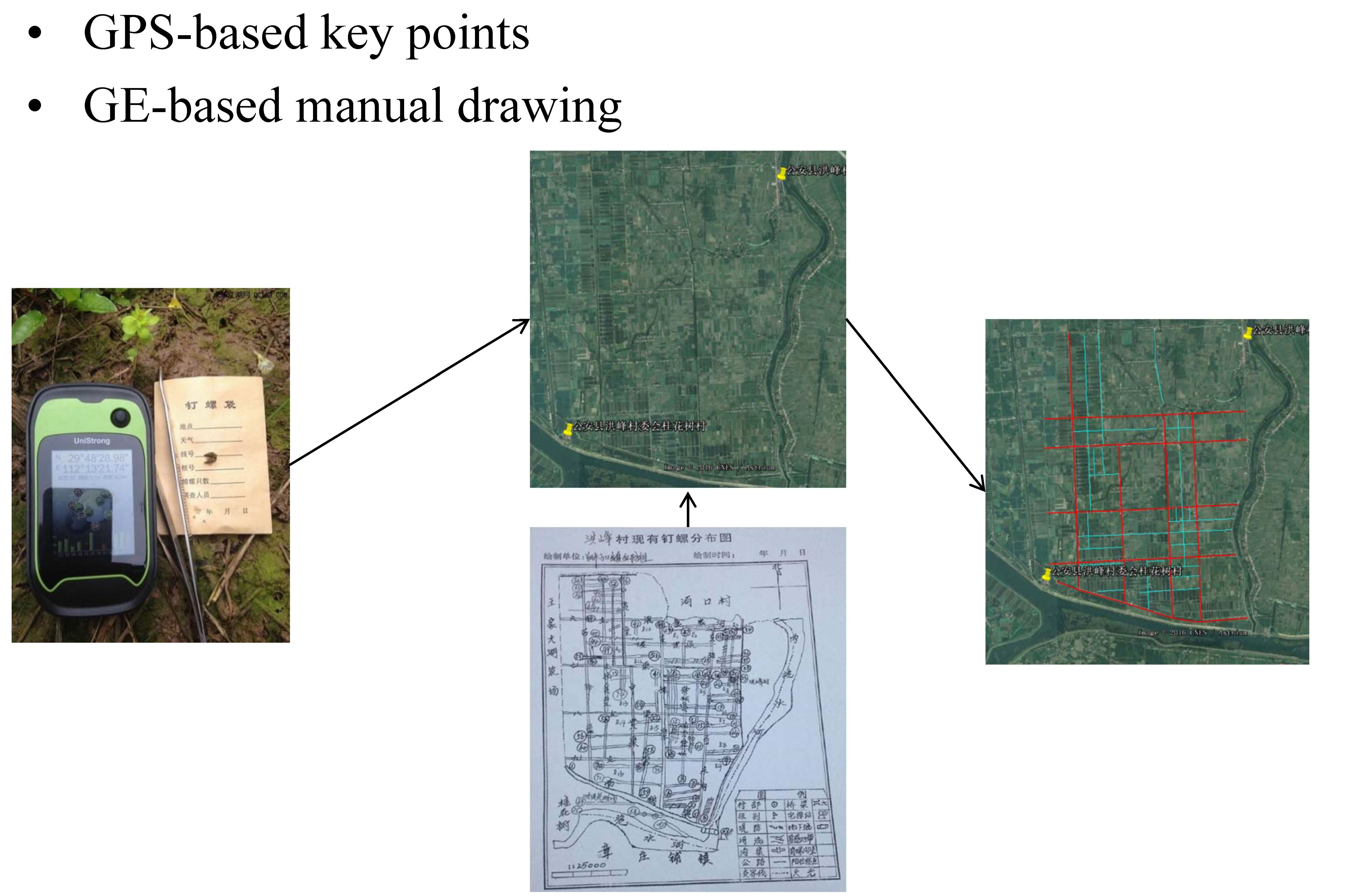

Supplement: Supplementary file 2 — Additional file 2: Figure S2. Digitalization flow of habitats based on historical data. [file 40249_2025_1281_MOESM2_ESM.jpg]

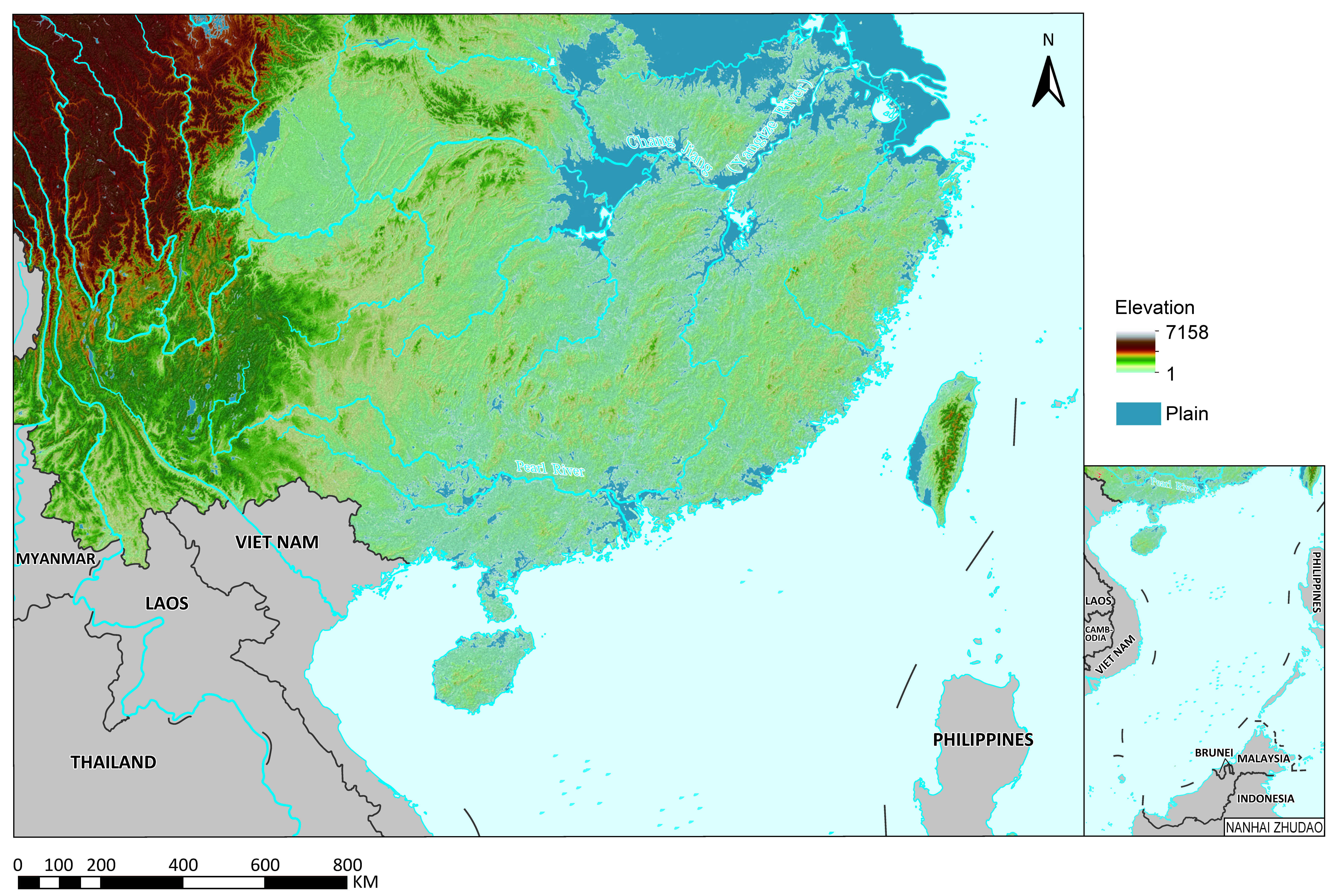

Supplement: Supplementary file 3 — Additional file 3: Figure S3: The plain areas (blue) distribution in the central and southern P.R. China. Map approval No.: GS (2025)0290. [file 40249_2025_1281_MOESM3_ESM.jpg]
